# Correlation from Undiluted Vitreous Cytokines of Untreated Central Retinal Vein Occlusion with Spectral Domain Optical Coherence Tomography

**DOI:** 10.2174/1874364101307010011

**Published:** 2013-03-08

**Authors:** MJ Koss, M Pfister, F Rothweiler, R Rejdak, R Ribeiro, J Cinatl, R Schubert, T Kohnen, FH Koch

**Affiliations:** 1 Department of Ophthalmology, Goethe University, Frankfurt am Main, Germany; 2 Doheny Eye institute, Los Angeles, USA; 3 Department of Virology, Goethe University, Frankfurt am Main, Germany; 4 Department of General Ophthalmology, Medical University in Lublin, Poland; 5 Medical Research Centre, Polish Academy of Sciences, Warsaw, Poland; 6 Department of Pediatric Pulmonology, Allergy and Cystic Fibrosis, Children's Hospital, Goethe University, Frankfurt am Main, Germany

**Keywords:** Vitreous samples, CRVO, VEGF, MCP-1, IL-6, CBA, SD-OCT.

## Abstract

**Purpose:**

To correlate inflammatory and proangiogenic key cytokines from undiluted vitreous of treatment-naïve central retinal vein occlusion (CRVO) patients with SD-OCT parameters.

**Methods:**

Thirty-five patients (age 71.1 years, 24 phakic, 30 nonischemic) underwent intravitreal combination therapy, including a single-site 23-gauge core vitrectomy. Twenty-eight samples from patients with idiopathic, non-uveitis floaterectomy served as controls. Interleukin 6 (IL-6), monocyte chemoattractant protein-1 (MCP-1), and vascular endothelial growth factor (VEGF-A) levels were correlated with the visual acuity (logMar), category of CRVO (ischemic or nonischemic) and morphologic parameters, such as central macular thickness-CMT, thickness of neurosensory retina-TNeuro, extent of serous retinal detachment-SRT and disintegrity of the IS/OS and others.

**Results:**

The mean IL-6 was 64.7pg/ml (SD ± 115.8), MCP-1 1015.7 ( ± 970.1), and VEGF-A 278.4 ( ± 512.8), which was significantly higher than the control IL-6 6.2 ± 3.4pg/ml (P=0.06), MCP-1 253.2 ± 73.5 (P<0.0000001) and VEGF-A 7.0 ± 4.9 (P<0.0006). All cytokines correlated highly with one another (correlation coefficient r=0.82 for IL-6 and MCP-1; r=0.68 for Il-6 and VEGF-A; r=0.64 for MCP-1 and VEGF-A). IL-6 correlated significantly with CMT, TRT, SRT, dIS/OS, and dELM. MCP-1 correlated significantly with SRT, dIS/OS, and dELM. VEGF-A correlated not with changes in SD-OCT, while it had a trend to be higher in the ischemic versus the nonischemic CRVO group (P=0.09).

**Conclusions:**

The inflammatory cytokines were more often correlated with morphologic changes assessed by SD-OCT, whereas VEGF-A did not correlate with CRVO-associated changes in SD-OCT. VEGF inhibition alone may not be sufficient in decreasing the inflammatory response in CRVO therapy.

## INTRODUCTION

Today, we can assess CRVO patients not only for changes in central macular thickness (CMT), but we can also conduct a detailed analysis of the neurosensory retina layers. Prognosis on visual acuity can be based upon the integrity of the IS/OS (photoreceptor inner and outer segments) and the external limiting membrane (ELM), which are important landmarks for good visual acuity rehabilitation. The development of subfoveal serous detachment seems thereby to be a potential negative clinical indicator [1-4]. In terms of reducing the frequent intravitreal reinjections, careful SD-OCT analysis allows for flexible anti-VEGF treatment, which has demonstrated significant functional and anatomic changes [5].

Macular edema secondary to central retinal vein occlusion (CRVO) occurs after multifactorial pathophysiologic changes, which affects intraocular cytokine levels [6-8]. Cytokines mediate between endothelial cells (EC) and inflammatory cells, which themselves interact with cytokine expression [9, 10]. The prolonged contact of ECs to proinflammatory cytokines might promote more thrombosis on top of the initial venous occlusion. Upregulated vascular endothelial growth factor (VEGF) is thereby a known chemoattractant cytokine for macrophages and leukocytes and thus plays an important role in the pathophysiologic dysbalance of CRVO [11].

Funk *et al*. recently demonstrated that anti-VEGF monotherapy has impact on the expression of VEGF and inflammatory markers, including interleukin 6 (IL-6) and monocyte chemoattractant protein 1 (MCP-1) [6]. IL-6 thereby is a major promoter of acute-phase proteins. Secondly it mediates by VEGF the change from acute to chronic inflammation, as it thus combines the inflammatory process with angiogenesis [12]. It could be demonstrated that the severity of macular edema (ME) is correlated with cytokine dysbalance [13].

Our group has previously described the rationale and the clinical outcome of a combination therapy including a core vitrectomy with the application of anti-VEGF agents and steroids and we have described the differences of intravitreal cytokines in different RVO categories [14,15]. Thereby we were able to acquire undiluted vitreous from CRVO patients before the drug injection and correlate the load of cytokines with detailed intraretinal layer changes assessed with SD-OCT.

## METHODS

This study was conducted after local institutional review board (IRB) approval. Following the sixth revision of the Declaration of Helsinki each participating patient consented to the study.

Patients were included with a CRVO associated significant macular edema (CSME) involving the fovea, but a macular thickness of not more than 1000 μm, which led to a visual acuity of not worse than 2.0 LogMAR. Not included were patients with CRVO associated complications, like iris rubeosis or neovascularization. Excluded were additionally patients with a history of previous intravitreal drug injections or surgery.

Early Treatment Diabetic Retinopathy Study (ETDRS) best-corrected visual acuity (BCVA) was assessed at 5 m with stopping at three out of five optotypes and presented as the logarithm of the minimum angle of resolution (LogMAR).

A SD-OCT (SD-OCT; 3D OCT-2000; Topcon, Tokyo, Japan) scan depth of 2.3 mm with a horizontal resolution of 20μm, and a longitudinal resolution of 5–6 μm was acquired at an A-scan speed of 27.000 A scans/second.

**Fig. (1):**
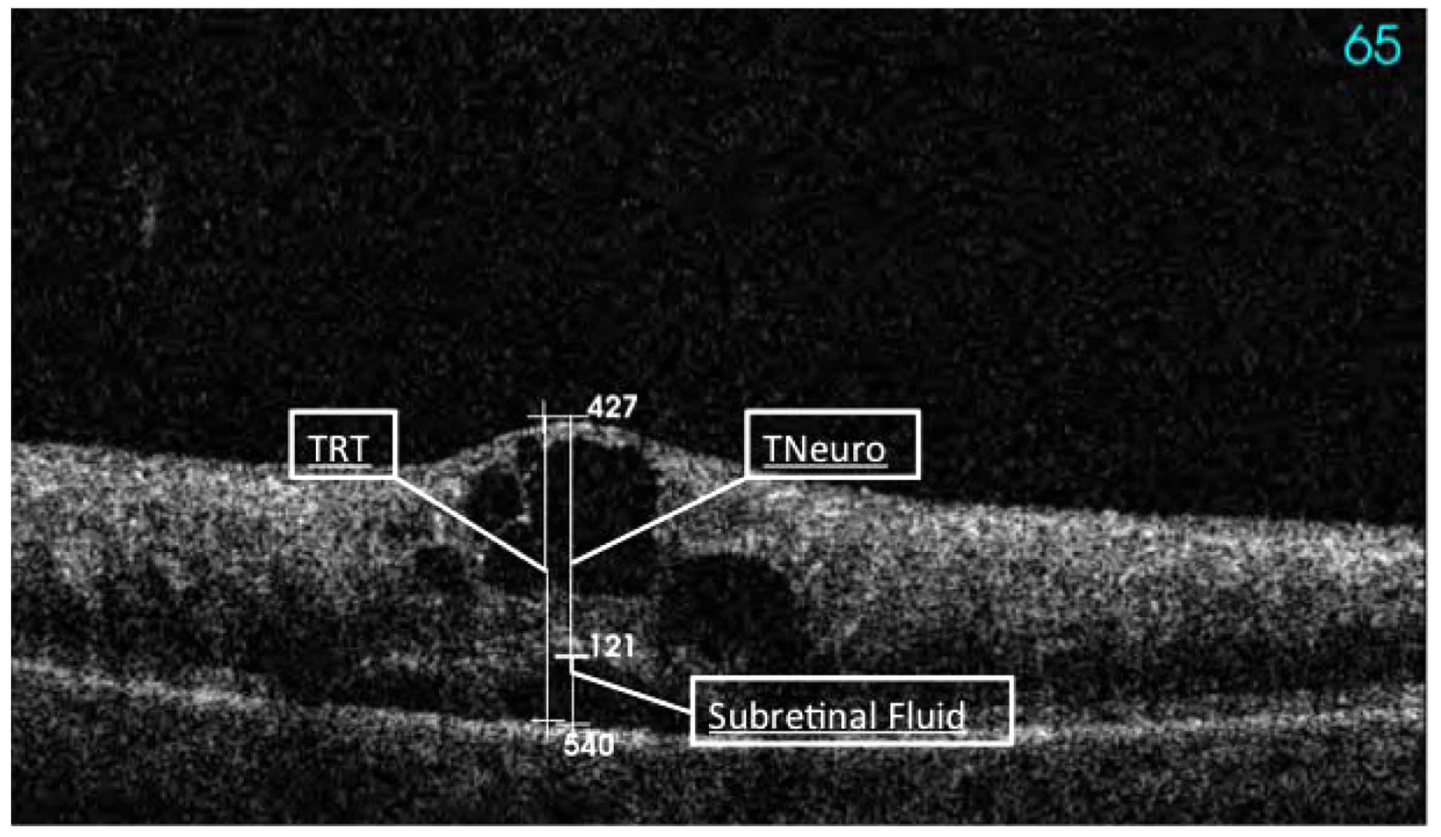
Cystoid macular edema secondary to a fresh CRVO assessed by SD-OCT with marked total retinal thickness (TRT), thickness neurosensory (TNeuro), and subretinal thickness marked as subretinal fluid (SRT) measured in µm

Consecutive sections and vertical and horizontal scans within the macular region were obtained by a well-trained OCT-certified technician (certification by the reading center, Vienna, Austria). Using OCT images, a standardized reading protocol was performed on OCT scans with a quality score over 16 dB, including six measurements (Fig. **1**):

1. Central macular thickness (CMT) was calculated as the distance of the inner limiting membrane (ILM) to the basal membrane (BM) of the retinal pigment epithelium (RPE) including all compartments in between.
2. Total retinal thickness (TRT) was defined as the biggest distance of the ILM to the BM of the RPE within the 3D scan field (127 A-scans), including
3. The Thickness of the neurosensory retina (TNeuro) and
4. The Subfoveal serous retinal thickness (SRT) and
5. The disintegrity of the inner and outer photoreceptor segments (dIS/OS)
6. The disintegrity of the external limiting membrane (dELM), both at the foveal region.

These measurements were reviewed with a caliper that was built into the software of the OCT machine by two retina specialists (M.P./F.K.) who were blinded to the visual acuity results and the results of the cytokine evaluation. Intergrader reliability (*κ*) was assessed with a *κ value *of 0.88 to 0.95. Additionally, the A-scans were evaluated for the occurrence of intraretinal cysts or hyperreflective spots. To exclude false positive occurrence of disrupted/ disintegrated IS/OS or ELM sections due to overlying cystic edema or intraretinal bleeding a-scans were scrolled through the macular region. Ischemic retinopathy was declared with >ten disc areas of nonperfusion with fluorescein angiography using the OIS WinStation (11K™, CCS Pawlowski GmbH, Jena, Germany). In case of early, fresh CRVO with extensive hemorrhages blocking retinal parts, FA was delayed until bleeding cleared up.

The surgical technique that yielded the sample collection was described earlier [16]. Briefly, 0.6 to 0.8ml of undiluted vitreous fluid from the mid- to posterior vitreous cavity were extracted by an assistant under the guidance of the surgeon, who controlled the vitrector with a headset and a magnifying 28-diopter lens.

Samples from idiopathic, non-uveitic floaterectomy served as controls. All samples were saved prior to drug application and frozen at –80°C.

The cytometric bead array system with flex sets (BD™, Heidelberg, Germany) was used to determine IL-6, MCP-1 and VEGF-A according to the manufacturer´s instruction manual including the measurement on a FACSArray™ Bioanalyzer (BD™, Heidelberg, Germany) with FCAP array software (BD™, Heidelberg, Germany). The data were saved in EXCEL^®^ (Microsoft Office 2010, Redmond, USA) and statistically analyzed with Bias^®^ software (Version 8.3.8, Epsilon, Darmstadt, Germany). The data had a non-parametrical distribution, which was checked with the David's test (error level of 5%), thus the Wilcoxon-Mann-Whitney test could be applied with a P value of <0.05.

Spearman rank correlation coefficient was used to examine the relationship between the influences of the cytokines on other parameters, like or changes in SD-OCT.

## RESULTS

### Patients

The CRVO group (14 men and 21 women) was aged 71.1 ± 11.7 years (mean ± standard deviation - SD), and the control group (12 men and 16 women) was aged 66.2 ± 7.9 years (P=0.89 and 0.17, respectively, Table **1**). The visual acuity was 1.28 ± 0.59 LogMAR in the CRVO group and 0.51 ± 0.22 in the control group (P<0.001). Twenty-four out of 35 in the CRVO group and 8 out 28 in the control group were phakic (P<0.005). Among the 35 CRVO patients, 30 were nonischemic. The duration of the CRVO was 7.4 ± 3.5 months for all CRVO patients and all were treatment naïve before study start. The patients were distributed in a subgroup of fresh CRVO with a duration of 5.1 ± 2.0 months (n=22) and a subgroup of old CRVO (n=13) with a duration of 11.2 ± 1.6 months after onset of the disease (P<0.001).

### SD-OCT

The mean absolute values ± SD with the 95% confidence interval (CI) of the SD-OCT measurements are depicted in μm in Table **2**. For all CRVO patients, the mean CMT was 549.3 ± 243.7 (CI 465.6-633μm), the TRT was 742.9 ± 256.3 (CI 654.8–830.9), the TNeuro was 555.5 ± 123.9 (CI 512.9–598.1), the SRT was 186.3 ± 149.4 (CI 134.9-237.6), the dIS/OS was 3252.1 ± 1321.3 (CI 2798.2–3705.9), and the dELM was 3649.8 ± 1214.7 (CI 3232.5–4067.1). In 34 out of 35 patients (97%), intraretinal cysts were noted, while in 8 out of 35 patients (23%), hyperreflective spots occurred. For the nonischemic and ischemic patients, higher values were observed for all SD-OCT parameters in the ischemic group, aside from subretinal thickness (SRT). This difference had a statistical trend for CMT (p=0.08), TNeuro (p=0.07), and the occurrence of intraretinal cysts (p=0.06). But regarding the SD-OCT parameters there was no statistical significant difference in the subgroups (old versus fresh and phakic versus pseudophakic).

### Correlation of SD-OCT

CMT correlated significantly (please see Table **3**) with TRT (r=0.83), with TNeuro (r=0.37), with SRT (r=0.42), with dIS/OS (r=0.49), with dELM (r=0.43), and inversely with intraretinal cysts (r= -0.52). TRT correlated significantly with TNeuro (r=0.55), with SRT (r=0.49), with dIS/OS (r=0.45), and with dELM (r=0.44). TNeuro and SRT correlated significantly with dIS/OS (r=0.41 and 0.51) and with dELM (r=0.45 and 0.54). DIS/OS and dELM correlated significantly with another (r=0.91). In total correlated CMT with six, dIS/OS and ELM with five, TRT with four, and TNeuro and SRT with two SD-OCT parameters.

### Vitreal Cytokine Levels

The mean IL-6 vitreal, MCP-1 and VEGF-A levels can be found in Table **2** and Fig. (**2**).

The IL-6 level was 66.8 ± 124.6 (CI 20.3–113.4) in the nonischemic CRVO group, the MCP-1 level was 1043.3 ± 1037.7 (655.8-1431), and the VEGF-A level was 277.7 ± 551.9 (71.6–484). In the ischemic group, the level of IL-6 was 51.7 ± 33 (CI 10.6–92.8, P=0.35), and the level of MCP-1 was 850.6 ± 390.4 (CI 365.8–1335, P=0.57). The VEGF-A level was higher in the ischemic group than in the nonischemic group, at 282.5 ± 165.9 (CI 76.4–488.6). The differences between the lens status was not significant. IL-6 was 71.7 ± 140.8 (CI -22.9–166.3) in the pseudophakic group and 61.5 ± 105.6 (CI 16.9–106, P=0.92) in the phakic group. The levels of MCP-1 were 1315.1 ± 1477.7 (CI 322.1–2308) and 878.6 ± 615.9 (CI 618–1139, P=0.43), while the level of VEGF-A was 249.5 ± 448.9 (CI -52.1–551.1) and 291.6 ± 548.2 (CI 60.1–523.1, P=0.34), respectively.

### Correlation Between Cytokines

In the CRVO group, all cytokines were positively correlated with one other. IL-6 was positively correlated (Table **3**) with both MCP-1 (r=0.82; P<0.0001) and VEGF-A (r=0.75; P<0.0001), MCP-1 was correlated with VEGF-A (r=0.62; P<0.0001). This was confirmed in the nonischemic CRVO subgroup analysis, where the IL-6 level was correlated with MCP-1 (r=0.83; P<0.0001) and VEGF-A (r=0.76; P<0.0001), and MCP-1 levels were correlated with VEGF-A (r=0.63; P<0.0002). In the ischemic subgroup, however, there was no significant correlation among the cytokines. Analysis of the pseudophakic versus the phakic subgroup yielded positive correlation coefficients for all three cytokines. In the pseudophakic group, the following correlations were observed: IL-6 and MCP-1: r=0.71, P<0.0001; IL-6 and VEGF-A: r=0.66, P<0.0004; MCP-1 and VEGF-A: r=0.51, P<0.01. In the phakic group, the following correlations were observed: IL-6 and MCP-1: r=0.98, P<0.0001; IL-6 and VEGF-A: r=0.99, P<0.0001; MCP-1 and VEGF-A: r=0.99, P<0.0001.

### Correlation of Cytokines with SD-OCT

IL-6 correlated more often with SD-OCT parameters than did MCP-1 (five times versus three times). IL-6 correlated significantly with CMT (r=0.53; P<0.001), TRT (r=0.49; P<0.003), SRT (r=0.41; P<0.02), dIS/OS (r=0.56; P<0.001), and dELM (r=0.58; P<0.003). MCP-1 correlated significantly with SRT (P<0.04); dIS/OS (r=0.38; P<0.02), and dELM (r=0.36; p<0.03). VEGF-A did not correlate with CRVO-associated changes in SD-OCT.

### Visual Acuity

Visual acuity was analyzed with LogMAR values and correlated to objective SD-OCT parameters and the vitreal cytokine values (Table **3**). There was no significant correlation between any of the parameters and visual acuity.

## DISCUSSION

We could demonstrate, that primarily the inflammatory cytokines were more often correlated with morphologic changes assessed by SD-OCT, whereas VEGF-A did not correlate with CRVO-associated changes in SD-OCT.

Recent studies with SD-OCT have shown that macular edema secondary to retinal vein occlusion is often characterized by numerous cystoid spaces and marked retinal swelling, especially in the outer retinal layers. We are not aware, that there is a correlation of SD-OCT parameters with intraocular cytokine levels published up to date. In our study, IL-6 correlated more often with SD-OCT parameters than MCP-1, whereas VEGF-A did not correlate with CRVO-associated changes in SD-OCT (Table **3**). CRVO macular edema is often accompanied by serous retinal detachment, which might occur as pointed or dome-shaped and might be associated with a poorer visual acuity prognosis [1]. The most suitable predictor of visual acuity, however, seems to be the integrity of the photoreceptor layer and the integrity of the external limiting membrane [3, 4]. Subretinal thickness, dIS/OS, and dELM were significantly correlated with the inflammatory marker IL-6 and not with VEGF-A. The visual acuity at the time of combination therapy was not correlated with objective SD-OCT parameters or the cytokines. Cystoid spaces themselves are often accompanied by hyperreflective spots, which might indicate chronicity [17]. Cystoid spaces and hyperreflective spots were correlated with central macular thickness and IL-6, which might underline chronicity. MCP-1, however, was not correlated with cystoid space or with hyperreflective spots. Generally, the role of MCP-1 in the pathophysiology of RVO is until today not well perceived. It has strong eosinophilic chemotactic properties and is crucial in monocyte recruitment at the endocytes, which supports the role of eosinophils in tissue remodeling [18].

**Fig. (3):**
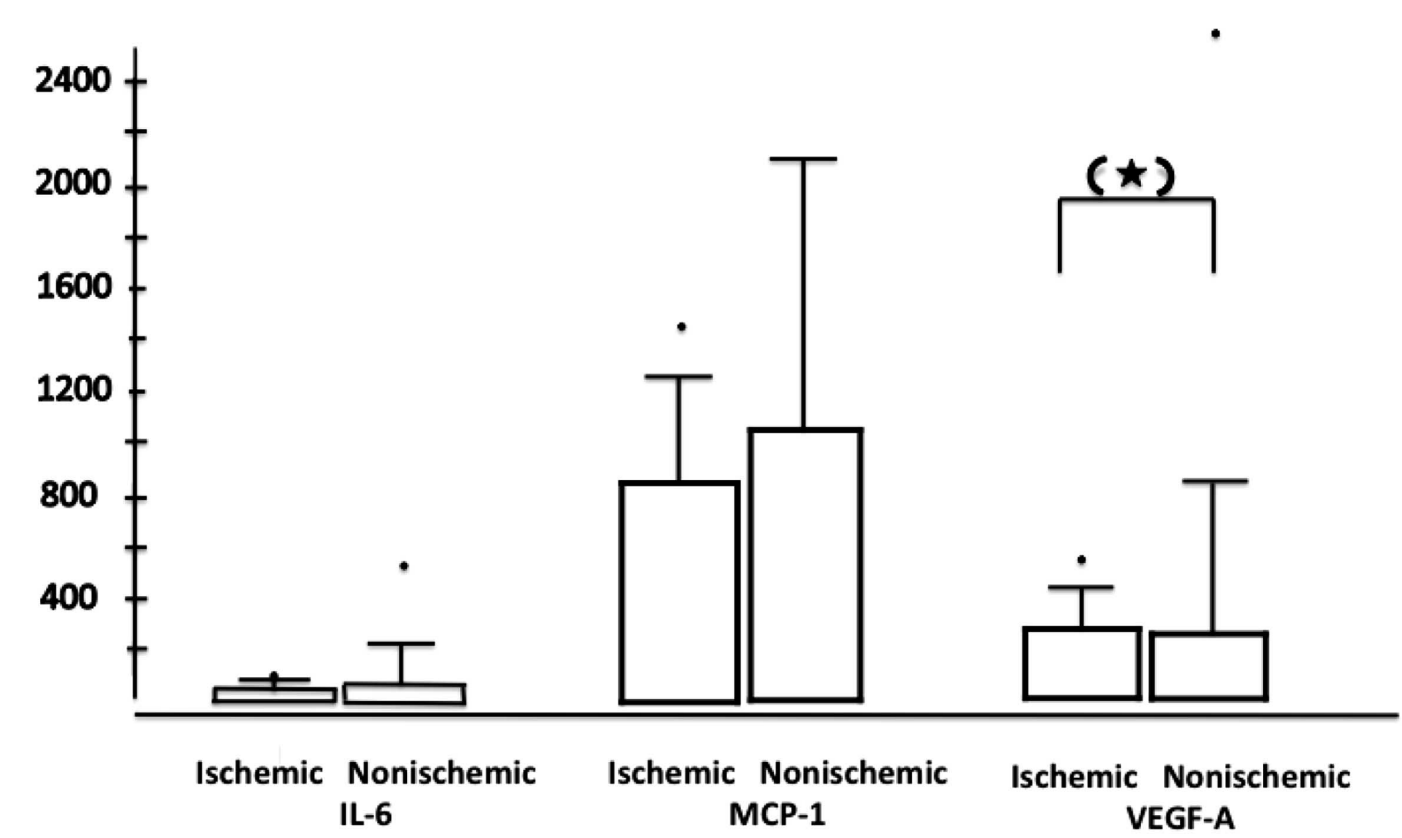
Bar plots with the values of cytokines (statistical extremes are marked with dots) for ischemic CRVO patients (left bar plots; n=15) versus non-ischemic CRVO patients (right bar plot; n=30); p=0.09 is marked with a star in brackets.

It is noteworthy that in our study all cytokines are positively correlated with each other (Table **3**), which was highly statistically significant. Because of one limitation of our study, the relatively small number of ischemic CRVO patients (n=5), we can only speculate about the implication of the results in terms of the comparison between ischemic versus nonischemic patients. While Noma *et al*. were able to demonstrate elevated IL-6 and VEGF levels in 18 ischemic CRVOs (versus 9 nonischemic CRVO) [7], we could not confirm this observation (Fig. **3**). There was a trend towards elevated VEGF-A levels in the ischemic group (p=0.09), but the correlation among the cytokines was insignificant, while there was a positive correlation between the cytokines in the nonischemic group. Based on routine clinical practice [10, 19] and previous preclinical publications [20, 21], it is likely that in CRVO with retinal ischemia, VEGF and IL-6 are both higher than in nonischemic CRVO. Cataract extraction after ischemic CRVO may imply a clinical risk of developing rubeosis iridis, but generally the implication of lens status (phakic or pseudophakic) is not well understood. We did not observe a significant difference between the two subgroups. The limitations of our study are a small sample size, which may have to oversee especially in the ischemic CRVO group significant differences.

In conclusion, we demonstrated significantly higher values of inflammatory and proangiogenic cytokines in eyes with “fresh”, treatment-naïve CRVO eyes as compared with control eyes. Although there was no correlation between morphologic SD-OCT parameters and visual acuity seven months after CRVO onset, inflammatory cytokines, IL-6 and MCP-1 were more often correlated with predictive morphologic changes (SRT, IS/OS, ELM), which are clinically important in visual acuity prognosis, than VEGF-A. VEGF-A had a tendency to be higher in the ischemic than in the nonischemic CRVO group.

## Figures and Tables

**Fig. (2):**
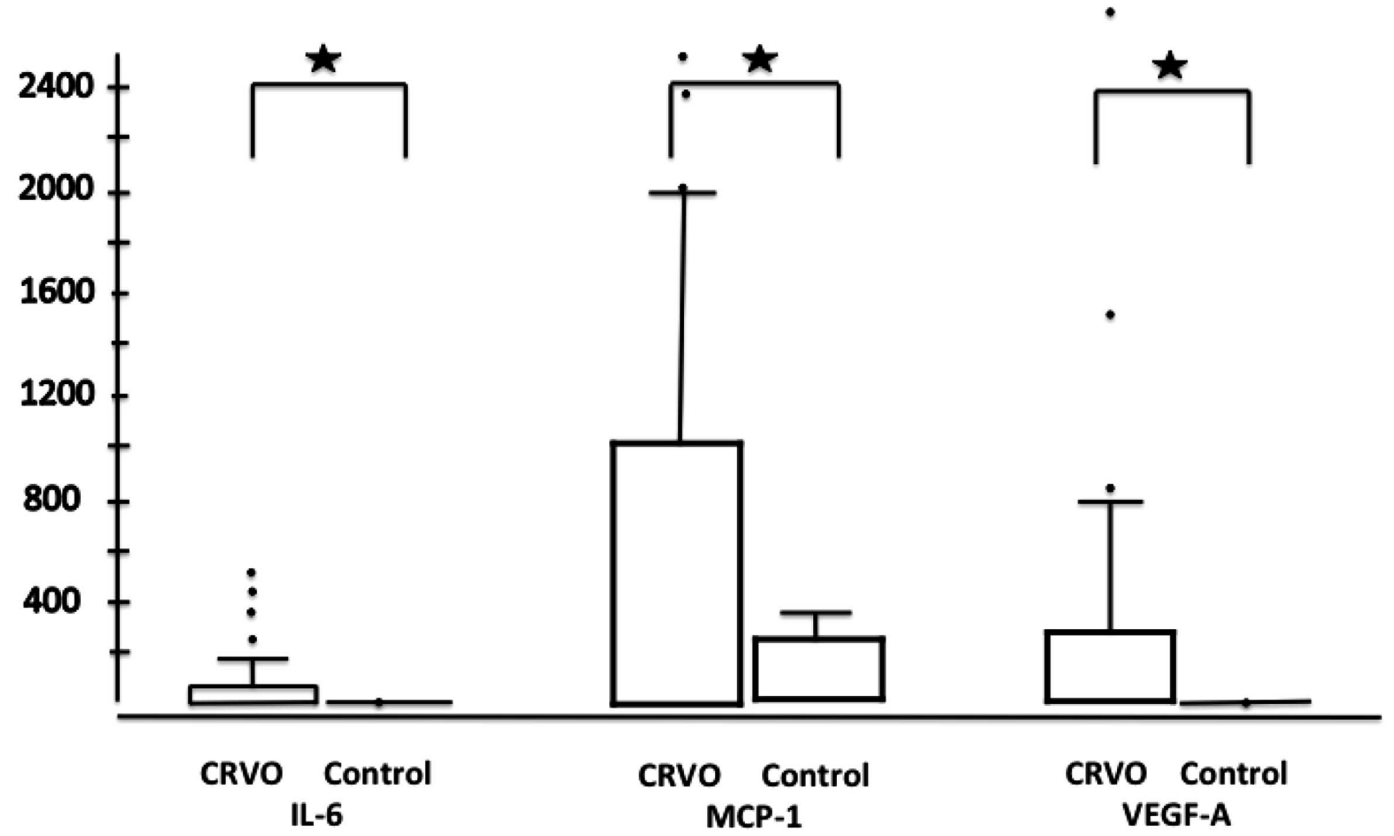
Bar plots with the values of cytokines (statistical extremes are marked with dots) for all CRVO patients (left bar plots; n=35) versus the control group (right bar plot; n=28); significant differences are noted with stars.

**Table 1.** Epidemiologics: Values in Mean ( ± Standard Deviation), Blood Pressure in mmHg, Duration of CRVO in Months, Ischemic Signs, Including Cotton Wools, Massive Intraretinal Haemorrhage, Enlarged Foveolar Avascular Zone, Capillary Drop-Outs, Area of Non-Perfusion > 5 Disc Areas, Differences are Calculated with Wilcoxon-Mann-Whitney Test

	CRVO	Control	P Value
**N**	35	28	
**Male/Female**	14/21	12/ 16	0.89
**Age in years**	71.1 ± 11.7	66.2 ± 7.9	0.17
**Blood Pressure**			
**Systolic**	141 ± 18	121 ± 10	*< 0.01*
**Diastolic**	83 ± 14	75 ± 8	0.09
**Hypertension**	24	5	*0.003*
**Drug therapy**	22	3	*0.004*
**VA in logMar**	1.28 ± 0.59	0.51 ± 0.22	*< 0.001*
**Pseudophakic/Phakic**	11/24	20/8	*< 0.005*
**Duration of CRVO**	7.4 ± 3.5		
**CRVO**			
**Type**	**Fresh**	**Older**	
**N**	22	13	
**Ischemic/Nonischemic**	3 / 19	2 / 11	0.96
**Duration of CRVO**	5.1 ± 2.0	11.2 ± 1.6	*< 0.001*

**Table 2.** Characteristics of the SD-OCT Measurements (Please See Fig. 1)

CRVO SD-OCT	All N=35			Nonischemic N=30	Ischemic N=5	P value	Pseudophakic N=11	Phakic N=24	P Value
**CMT**	549.3 ± 243.7 (465.6-633)			521.8 ± 238.9 (432.5-611)	714.8 ± 225.8 (434.5-995.1)	0.08	475.9 ± 221.4 (327.2-624.7)	583 ± 250.5 (477.2-688.8)	0.17
**TRT**	742.9 ± 256.3 (654.8-830.9)			717.6 ± 245.7 (625.8-809.4)	894.6 ± 295.3 (527.9-1261.3)	0.15	669.7 ± 292 (473.5-865.9)	776.4 ± 237.3 (676.2-876.6)	0.15
**TNeuro**	555.5 ± 123.9 (512.9-598.1)			538.1 ± 119.9 (493.4-582.9)	659.8 ± 101.9 (533.2-786.4)	0.07	562.9 ± 154.4 (459.2-666.6)	552.1 ± 110.8 (505.3-598.9)	0.84
**SRT**	186.3 ± 149.4 (134.9-237.6)			192.9 ± 146.2 (138.3-247.5)	146.6 ± 180.1 (-77.1-370.2)	0.42	162.8 ± 134.9 (72.2-253.5)	197.1 ± 157.1 (130.7-263.4)	0.47
**dIS/OS**	3252.1 ± 1321.3 (2798-3706)			3143.8 ± 1333 2646-3642)	3902 ± 1158.1 (2464-5340)	0.24	3210.3 ± 1716.9 (2057-4364)	3271.3 ± 1139.3 (2790-3752)	0.93
**dELM**	3649.8 ± 1214.7 (3233-4067)			3525.3 ± 1234.9 (3064-3986)	4396.8 ± 820.2 (3378-5415)	0.24	3389.5 ± 1560 (2341-4438)	3769.1 ± 1037.1 (3331-4207)	0.49
**Cysts**	34			30	4	0.06	11	23	0.16
**HR-Spots**	8			8	0	0.37	3	5	0.79
CRVO Cytokines									
	All	Control	P value	Nonischemic	Ischemic	P value	Pseudophakic	Phakic	P value
**IL-6**	64.7 ± 115.8 (24.9-104.4)	6.2 ± 3.4 (4.9-7.5)	0.06	66.8 ± 124.6 (20.3-113.4)	51.7 ± 33 (10.6-92.8)	0.35	71.7 ± 140.8 (-22.9-166.3)	61.5 ± 105.6 (16.9-106)	0.92
**MCP-1**	1015.8 ± 970.1 (682.5-1349)	253 ± 74 (225-282)	0.001	1043.3 ± 1037.7 (655.8-1431)	850.6 ± 390.4 (365.8-1335)	0.57	1315.1 ± 1477.7 (322.3-2308)	878.6 ± 615.9 (618-1139)	0.43
**VEGF-A**	278.4 ± 512.8 (102.2-454.5)	7 ± 4.9 (5.1-8.9)	0.001	277.7 ± 551.9 (71.6-484)	282.5 ± 165.9 (76.4-488.6)	0.09	249.5 ± 448.9 (-52.1-551.1)	291.6 ± 548.2 (60.1-523.1)	0.34

All measurements are depicted as mean values ± standard deviation (SD) in μm. The 95% confidence interval values are depicted in brackets; CMT=central macular thickness, TRT=total retinal thickness; TNeuro=thickness neurosensorium; SRT=subretinal thickness; dIS/OS= discontinued inner and outer photoreceptor segment band in the fovea; dELM=discontinued external limiting membrane band; Cysts=intraretinal cysts; HR spots= hyperreflective spots. The absolute mean values of the vitreous cytokines ± standard deviation (SD) are in pg/ml with the 95% confidence interval values in brackets.

**Table 3.** Spearman Rang Correlation Matrix

	VA	CMT	TRT	TNeuro	SRT	dIS/OS	dELM	Cysts	Ischemic	IL-6	MCP-1	VEGF-A
**VA**		0.15 (0.39)	0.14 (0.42)	-0.04 (0.80)	0.27 (0.11)	0.26 (0.13)	0.16 (0.35)	-0.04 (0.81)	-0.27 (0.12)	0.25 (0.15)	0.26 (0.12)	0.15 (0.8)
**CMT**			0.83 (0.001)	0.37 (0.03)	0.42 (0.01)	0.49 (0.003)	0.43 (0.01)	-0.52 (0.002)	-0.30 (0.08)	0.53 (0.001)	0.21 (0.23)	0.33 (0.05)
**TRT**				0.55 (0.001)	0.49 (0.003)	0.45 (0.01)	0.44 (0.01)	-0.22 (0.21)	-0.25 (0.14)	0.49 (0.003)	0.29 (0.10)	0.33 (0.06)
**TNeuro**					0.18 (0.30)	0.41 (0.02)	0.45 (0.01)	-0.02 (0.90)	-0.32 (0.07)	0.18 (0.30)	0.10 (0.57)	0.18 (0.30)
**SRT**						0.51 (0.002)	0.54 (0.001)	-0.13 (0.47)	0.15 (0.39)	0.41 (0.02)	0.35 (0.04)	0.05 (0.79)
**dIS/OS**							0.91 (0.001)	-0.28 (0.11)	-0.21 (0.21)	0.56 (0.001)	0.30 (0.08)	0.20 (0.24)
**dELM**								-0.28 (0.11)	-0.21 (0.21)	0.58 (0.003)	0.38 (0.02)	0.35 (0.04)
**Cysts**									0.40 (0.02)	-0.12 (0.50)	0.19 (0.28)	-0.02 (0.90)
**Ischemic**										-0.17 (0.33)	-0.11 (0.55)	-0.28 (0.10)
**IL-6**											0.82 (0.001)	0.68 (0.001)
**MCP-1**												0.64 (0.001)
**VEGF-A**												

Subjective (logMar-VA) and objective (SD-OCT) values are correlated with each other and with the values for vitreal cytokines (IL-6, MCP-1, and VEGF-A). The results are represented with the correlation coefficient r (p values are in brackets). The statistically significant correlations are depicted as bold results.
